# Supplementary material for: LNX1 Contributes to Cell Cycle Progression and Cisplatin Resistance
Source: Cancers (Basel). 2021 Aug 12;13(16):4066. doi: 10.3390/cancers13164066 (PMC8394373; doi:10.3390/cancers13164066)
Supplement: Supplementary file 1 [file cancers-13-04066-s001.zip › cancers-1321219-supplementary.pdf]

Supplementary Materials: *LNX1* Contributes to Cell Cycle Progression and Cisplatin Resistance

Minsu Jang, Rackhyun Park, Yea-In Park, Yeonjeong Park, Jin I. Lee, Sim Namkoong, Eun-Ju Lee and Junsoo Park

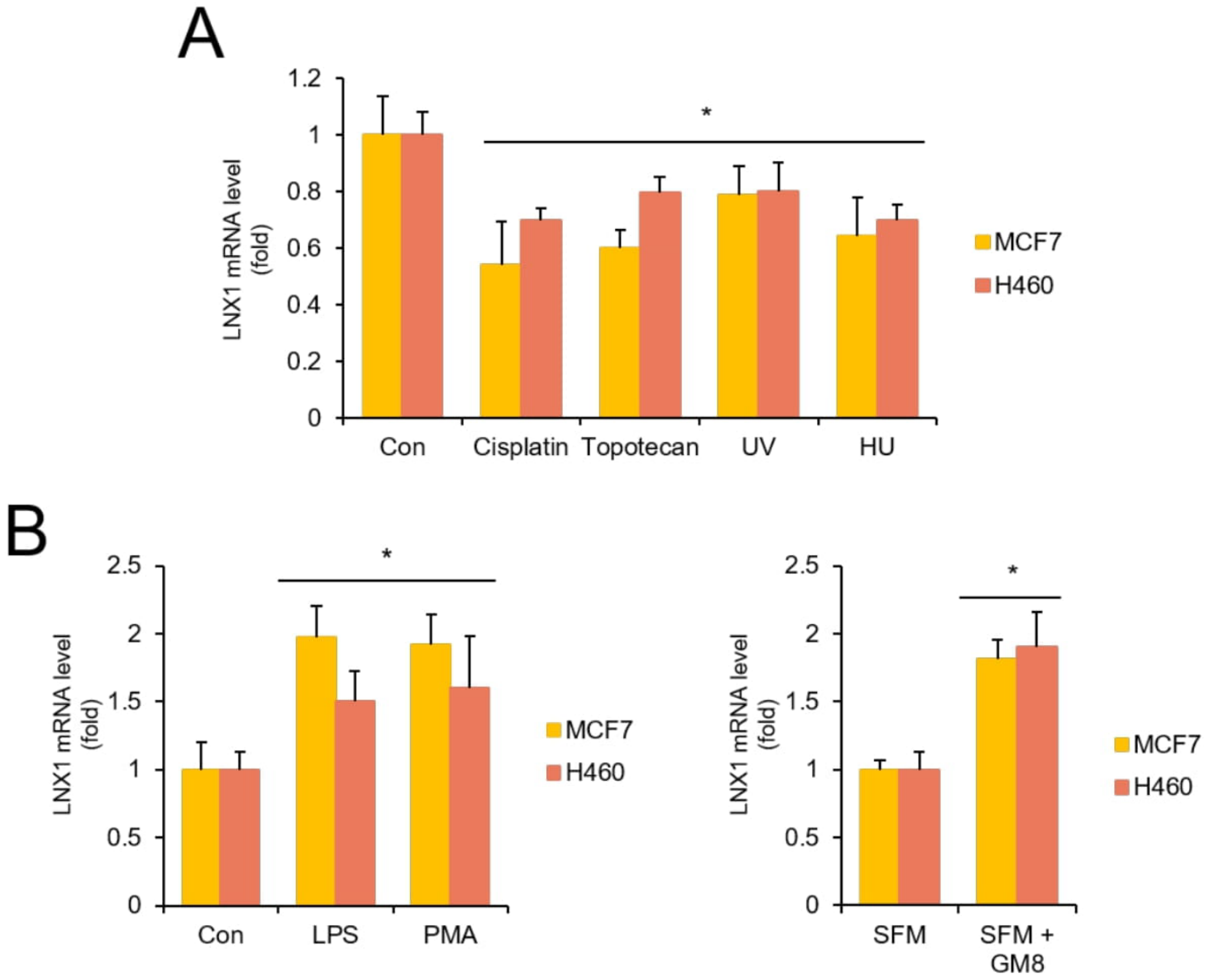

Figure S1. DNA damage and cell growth were associated with expression of *LNX1* in MCF7 and H460 cells.(**A**) Each cell was treated with cisplatin (10 μg/mL), topotecan (0.1 μM) and Hydroxyrurea (1 μM) or irradiated with UV (50 mJ/cm²). (**B**) Each cell was treated with LPS (100 ng/mL), PMA (200 nM) or incubated with serum-free medium for 24 h. serum-free medium was replaced to growth media and cells were incubated for 8 h. Expression levels were calculated by quantitative real-time PCR analysis. The results are represented as relative mRNA levels. Control vs. Drug-treated cells, *: $p < 0.05$.

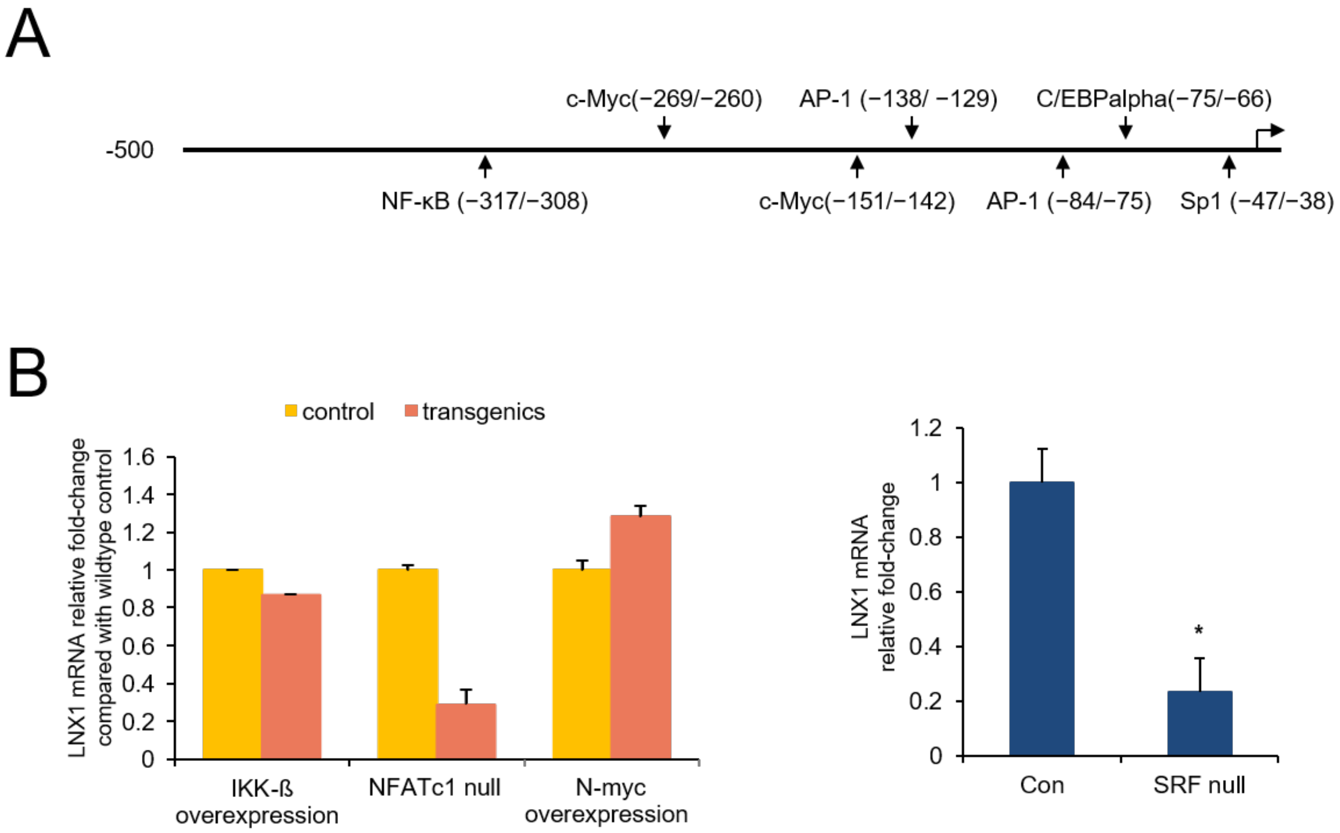

Figure S2. Several oncogenic transcription factors are involved with the expression of *LNX1*. (**A**) Potential transcription factor binding sites within the *LNX1* gene promoter were screened using the AliBaba 2.1 program (www.gene-regulation.com). The program searches putative transcription binding sites against the TRANSFAC database. (**B**) Serum response factor, *NFATc1*, *IKK-ß* and *N-myc* contribute to the expression of *LNX1*. Expression profiles from the GSE13333, GSE17511, GSE37219, and GSE6077 datasets were obtained from the Gene Expression Omnibus. The expression of LNX1 was analyzed, and the relative expression levels are shown in the graph. Wild type-mice vs. SRF deficient mice, *: $p < 0.05$.

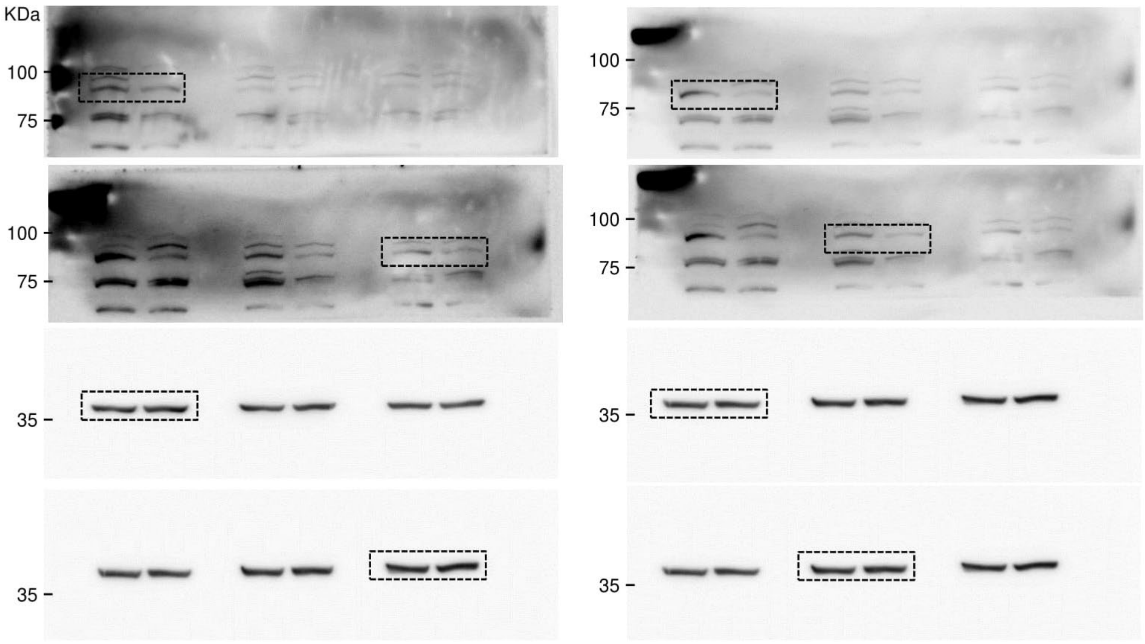

Figure 1C

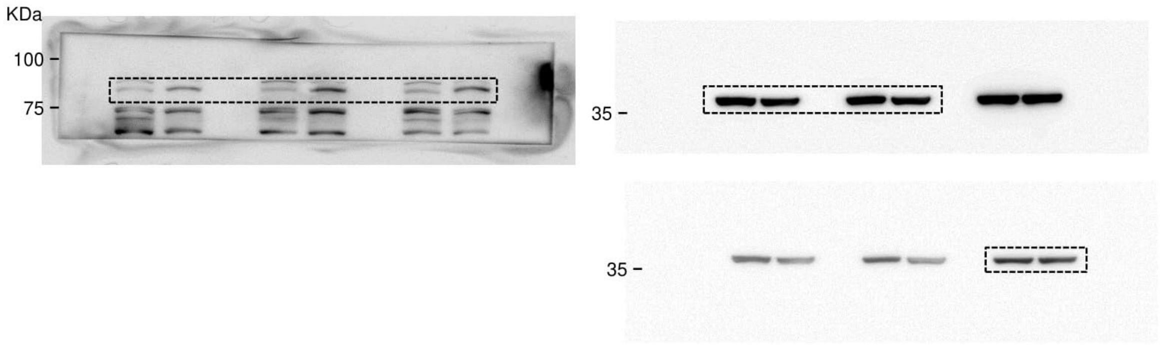

Figure 2C

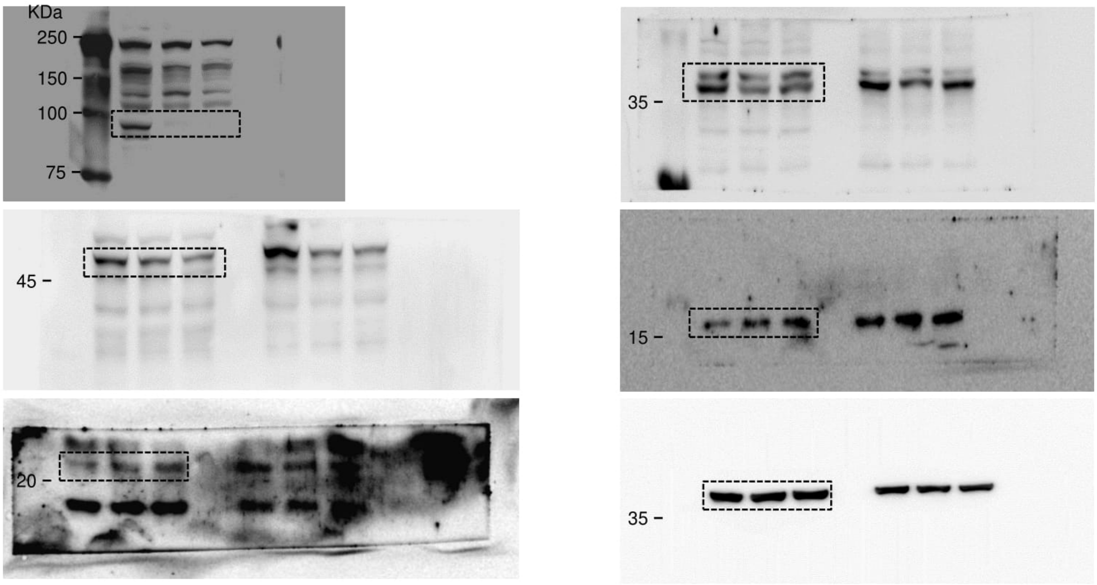

Figure 4A

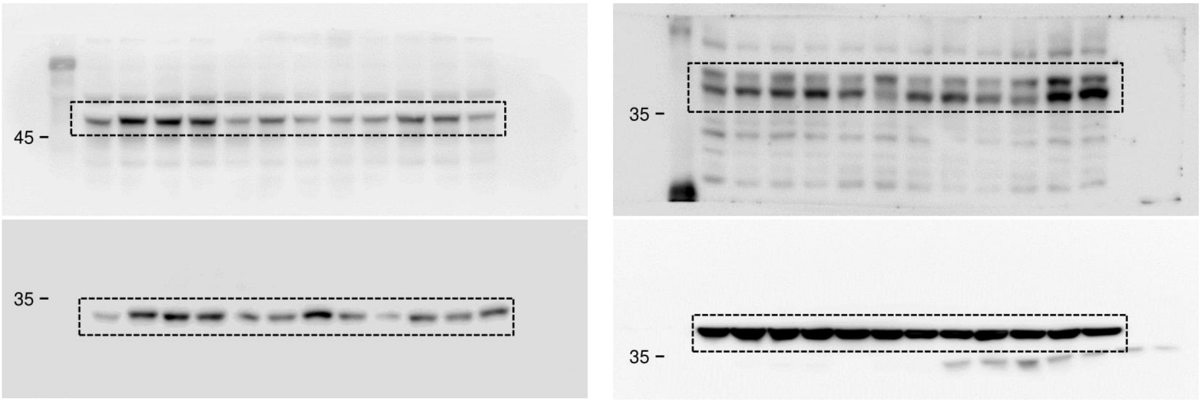

Figure 4C

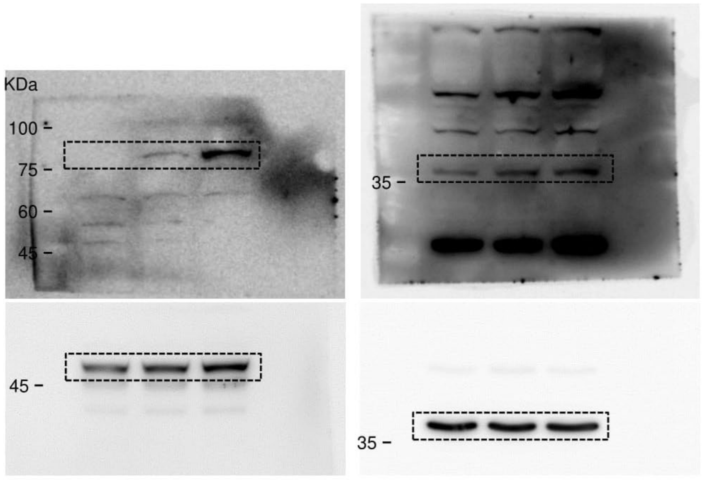

Figure 5A

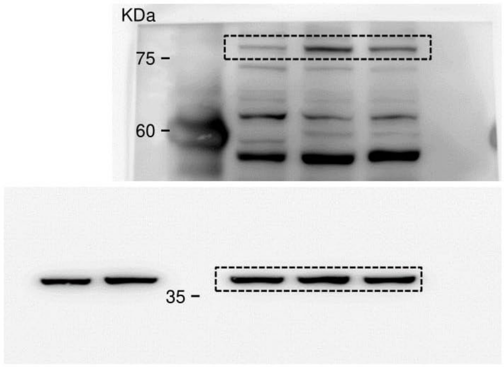

Figure 5D

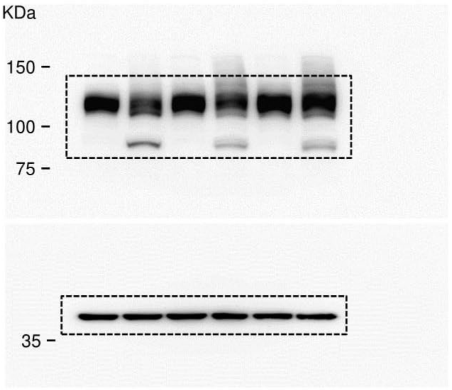

Figure 6E

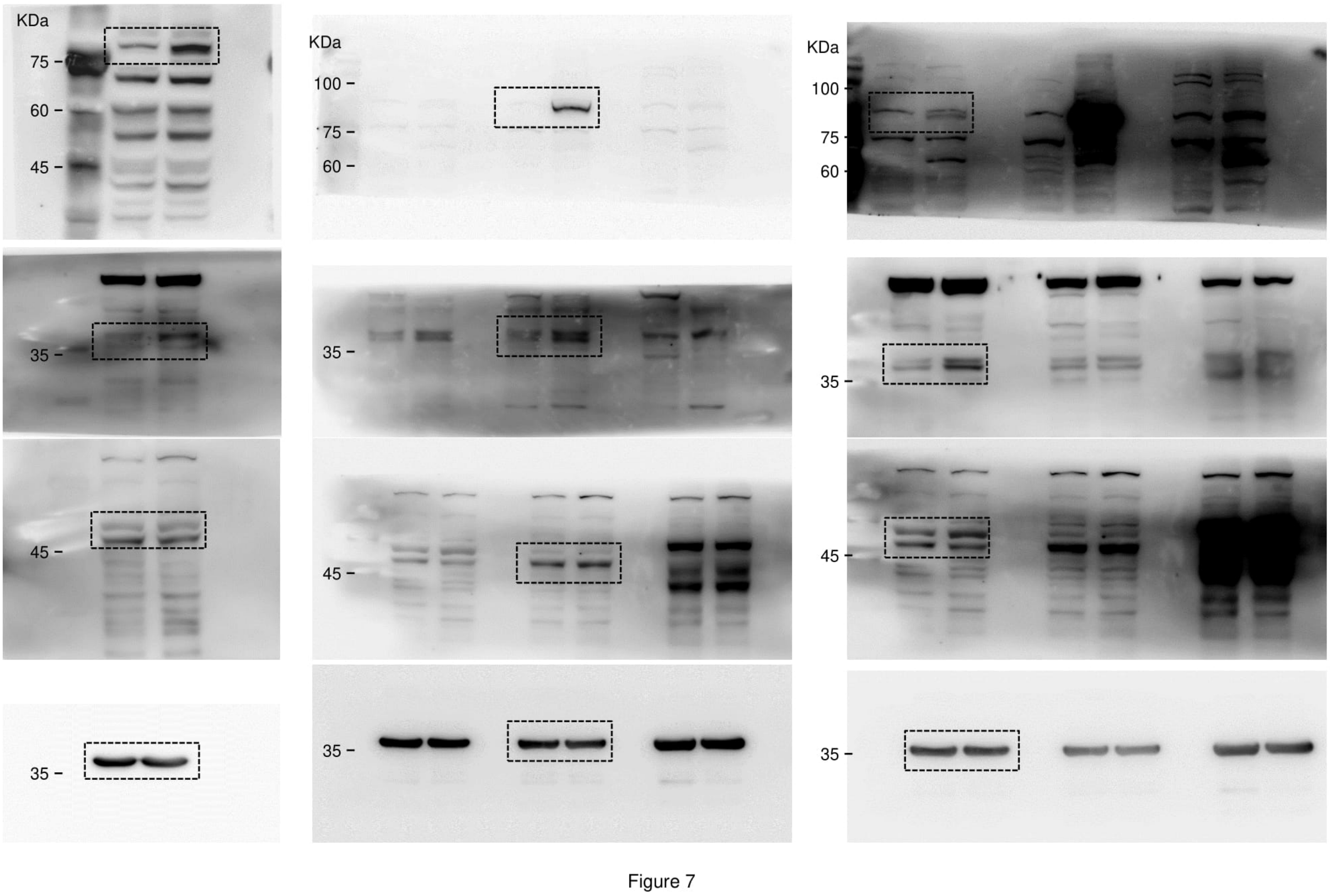

Figure S3. Full length blots cropped for representative figures.
